# Designed SARS‐CoV‐2 receptor binding domain variants form stable monomers

**DOI:** 10.1002/biot.202100422

**Published:** 2022-02-03

**Authors:** Miriam Klausberger, Nikolaus F. Kienzl, Gerhard Stadlmayr, Clemens Grünwald‐Gruber, Elisabeth Laurent, Katharina Stadlbauer, Florian Stracke, Klemens Vierlinger, Manuela Hofner, Gabriele Manhart, Wilhelm Gerner, Florian Grebien, Andreas Weinhäusel, Lukas Mach, Gordana Wozniak‐Knopp

**Affiliations:** ^1^ Institute of Molecular Biotechnology, Department of Biotechnology University of Natural Resources and Life Sciences (BOKU) Vienna Austria; ^2^ Institute of Plant Biotechnology and Cell Biology, Department of Applied Genetics and Cell Biology University of Natural Resources and Life Sciences (BOKU) Vienna Austria; ^3^ Christian Doppler Laboratory for Innovative Immunotherapeutics University of Natural Resources and Life Sciences (BOKU) Vienna Austria; ^4^ Institute of Biochemistry, Department of Chemistry and BOKU Core Facility Mass Spectrometry University of Natural Resources and Life Sciences (BOKU) Vienna Austria; ^5^ BOKU Core Facility Biomolecular & Cellular Analysis University of Natural Resources and Life Sciences (BOKU) Vienna Austria; ^6^ Competence Unit Molecular Diagnostics, Center for Health and Bioresources Austrian Institute of Technology Vienna Austria; ^7^ Institute of Medical Biochemistry University of Veterinary Medicine Vienna Austria; ^8^ Institute of Immunology University of Veterinary Medicine Vienna Austria

**Keywords:** antibody assay validation, antigen stability, COVID‐19, receptor binding domain, recombinant expression

## Abstract

The receptor binding domain (RBD) of the SARS‐CoV‐2 spike (S)‐protein is a prime target of virus‐neutralizing antibodies present in convalescent sera of COVID‐19 patients and thus is considered a key antigen for immunosurveillance studies and vaccine development. Although recombinant expression of RBD has been achieved in several eukaryotic systems, mammalian cells have proven particularly useful. The authors aimed to optimize RBD produced in HEK293‐6E cells towards a stable homogeneous preparation and addressed its O‐glycosylation as well as the unpaired cysteine residue 538 in the widely used RBD (319‐541) sequence. The authors found that an intact O‐glycosylation site at T323 is highly relevant for the expression and maintenance of RBD as a monomer. Furthermore, it was shown that deletion or substitution of the unpaired cysteine residue C538 reduces the intrinsic propensity of RBD to form oligomeric aggregates, concomitant with an increased yield of the monomeric form of the protein. Bead‐based and enzyme‐linked immunosorbent assays utilizing these optimized RBD variants displayed excellent performance with respect to the specific detection of even low levels of SARS‐CoV‐2 antibodies in convalescent sera. Hence, these RBD variants could be instrumental for the further development of serological SARS‐CoV‐2 tests and inform the design of RBD‐based vaccine candidates.

## Graphical Abstract and Lay Summary

The receptor binding domain (RBD) of the SARS‐CoV‐2 spike protein is a prime target of virus‐neutralizing antibodies present in convalescent sera of COVID‐19 patients, and considered a key antigen for immunosurveillance studies and vaccine development. The authors show that deletion or substitution of the unpaired cysteine residue C538 increases the tendency of RBD to form stable monomers and such mutants are suitable for serum screening . Further, intact O‐glycosylation at T323 position was found also highly relevant for the stability of RBD.

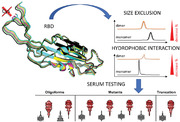

**Abbreviations**
ACE2: angiotensin‐converting enzyme 2
AUC: area under the curve
COVID‐19: coronavirus disease 2019
Cp: heat capacity
DSC: differential scanning calorimetry
ELISA: enzyme‐linked immunosorbent assay
Fc: fragment crystallizing
HIC: hydrophobic interaction chromatography
HPLC: high performance liquid chromatography
IgG: immunoglobulin G
IgM: immunoglobulin M
MES: 2‐(N‐morpholino)ethanesulfonic acid
MFI: median fluorescence intensity
MWCO: molecular weight cut‐off
NTA: nitrilotriacetic acid
PBS: phosphate buffered saline
PEI: polyethyleneimine
RBD: receptor binding domain
RBM: receptor binding motif
ROC: receiver‐operating characteristic curve
RT: room temperature
SARS‐CoV‐2: severe acute respiratory syndrome coronavirus type 2
SASA: solvent‐accessible surface area
SDS‐PAGE: sodium dodecyl sulfate polyacrylamide gel electrophoresis
SEC: size‐exclusion chromatography
*T*_M_
: midpoint transition temperature

## 1 INTRODUCTION

In the SARS‐CoV‐2 outbreak of 2019, there was an urgent need to rapidly develop reagents that would serve as components of serological diagnostic tests.^[^ ^1^, ^2^ ^]^ The detection of specific antibodies in serum samples complements PCR‐based infection diagnosis in delivering evidence of past infections and allows the determination of the extent of vaccination responses. Molecules selected for diagnostic purposes typically represent parts of the respective virus coat protein carrying antigenic determinants. Apart from exhibiting exquisite specificity, these proteins should feature cost‐effective production yields and high biophysical stability.^[^ ^3^ ^]^ The critical point of infection with SARS‐CoV‐2 has early been established as the interaction of the viral S‐protein with its cellular receptor angiotensin‐converting enzyme 2 (ACE2).^[^ ^4^ ^]^ This complex viral protein is conformationally highly variable,^[^ ^5^ ^]^ and its sequence contains up to 22 potentially used N‐linked glycosylation sites, which renders it a challenging target for heterologous expression.^[^ ^6^ ^]^ The amino acid residues in the S‐protein that are actually involved in the interaction with ACE2 could be narrowed down to a region referred to as the receptor binding motif (RBM),^[^ ^7^ ^]^ which is part of an independently folding and mostly solvent‐exposed fragment termed the receptor binding domain (RBD).^[^ ^8^ ^]^ Importantly, the RBD can be expressed at much higher yields (up to 90 mg L^–1^)^[^ ^9^ ^]^ than the full‐length S‐protein ectodomain (1–2 mg L^–1^) in various eukaryotic expression systems^[^ ^10^ ^]^ and therefore appears to be a promising vaccine candidate.^[^ ^11^ ^]^ The first variants of RBD produced for structural characterization and diagnostic applications consisted of S‐residues 319–541 (numbering as in PDB: 6VXX). As C538 forms a disulfide bond with C590 in the full‐length S‐protein (PDB: 6VXX),^[^ ^12^ ^]^ the unpaired cysteine in recombinant RBD forms could lead to inadvertent dimer formation. The resulting protein preparations might thus contain multiple oligomeric species with distinct sets of properties. Dimers and higher‐order multimers can skew the results of serological tests as well as the outcome of antigen quality assessments such as receptor‐binding studies by surface plasmon resonance spectroscopy or biolayer interferometry. Typically, multimeric protein forms are removed by preparative size‐exclusion chromatography (SEC), hence a lower proportion of dimeric species in the starting material could lead to a higher yield of the final product.

From the collection of expression systems so far used to produce SARS‐CoV‐2 RBD (which includes prokaryotic,^[^ ^13^ ^]^ yeast,^[^ ^14^ ^]^ plant,^[^ ^15^ ^]^ insect^[^ ^1^, ^10^ ^]^ and mammalian cell systems^[^ ^10^, ^16^ ^]^), human embryonic kidney HEK293 cells scored as the most potent system for production of an antigen suitable for diagnostic procedures. HEK293‐produced RBD is typically N‐glycosylated at two sites, displaying complex‐type N‐glycosylation at N331 and N343 (PDB: 6M17).^[^ ^8^ ^]^ The “glycan shield” modulates the structural dynamics of RBD and is hence critical for viral infectivity.^[^ ^17^ ^]^ Glycan moieties at these two positions can critically influence the expression and folding of recombinant RBD, which was demonstrated for RBD produced in bacteria^[^ ^13^ ^]^ and yeast,^[^ ^14^ ^]^ where lack of glycosylation or hyper‐mannosylation respectively, influenced the quaternary status of the protein and its antigenic properties. Additional heterogeneity of recombinant RBD can arise from O‐glycosylation. Up to six such glycosylation sites were found to be utilized in HEK293‐expressed RBD, although only T323 showed substantial occupancy.^[^ ^18^ ^]^

In the present work, we aimed to produce RBD variants that are less prone to form dimers, but are not negatively impacted in expression, thermostability, storage stability and their receptor and antibody binding properties. We hypothesized that this could be achieved by deletion or substitution of the unpaired cysteine C538. We also addressed the functional relevance of RBD O‐glycosylation by replacing residue T323 with alanine to obliterate the major O‐glycosylation site. Validation of respective protein sequences and analysis of N‐ and O‐glycosylation patterns was performed using mass spectrometry‐based peptide mapping. We also examined the quaternary status, stability, and hydrophobic properties of the RBD mutants, and determined the extent of their reactivity with ACE2 in vitro. The suitability of the resulting RBD variants for serological diagnosis was assessed with a large cohort of sera obtained from COVID‐19 patients and non‐infected individuals.

## 2 EXPERIMENTAL SECTION

### 2.1 Computational

The PDB structure PDB:6VXX served as basis for the design of all RBD mutants. Removing the amino acid sequence C‐terminal to residue K537, which could contribute to the stability of the domain by forming a salt bridge with E324, was the rationale for design of the shortened mutant tRBD (residues 319–537). Alternatively, the cysteine residue at position 538 was substituted with A, G, P, or S in RBD (319‐541). Models were constructed using the FoldX plugin^[^ ^19^ ^]^ in YASARA,^[^ ^20^ ^]^ using the site mutagenesis tool with two energy minimization rounds at 298 K, pH 7, ionic strength 500 and van der Waals’ design of 2. The PositionScan function of the FoldX algorithm was applied to examine any possible destabilization. The solvent‐accessible surface area (SASA) of C538 or its substitutions was determined using the PyMOL get_area function with solvent density set to 3.

### 2.2 Recombinant protein production

The RBD sequence used corresponded to the genomic sequence of the Wuhan‐Hu‐1 isolate (GenBank: MN908947.3).^[^ ^21^ ^]^ Mutants were produced in pTT28 vectors (Canadian National Research Council, CNRC) and were obtained using either directional cloning between the *Nhe*I and *BstE*II sites or site‐directed mutagenesis with the Quikchange Lightning Mutagenesis kit (Agilent Technologies) using oligonucleotides listed in Table S1, Supporting Information. High‐quality plasmid DNA was prepared using the Nucleobond Xtra Midi kit (Macherey‐Nagel) and sterilized using Ultrafree‐MC centrifugal filter units (Merck Millipore). Ten micrograms DNA, resuspended in 1 ml supplemented F17 medium (F17 with 4 mM L‐glutamine, 0.1% v/v Pluronic F68 and 25 μg ml^–1^ G‐418) (Thermo Fisher Scientific), were combined with 20 μg polyethyleneimine (PEI) in 1 ml supplemented F17 for 15 min at room temperature (RT), and then this mixture was added dropwise per 10 ml of HEK293‐6E cells (CNRC) at a density of 1.5–2.0 × 10^6^/ml. Cells were kept on an orbital shaker at 130 rpm and 37°C, in a humidified atmosphere containing 5% CO_2_, in supplemented F17 medium and fed with 0.25% v/v tryptone TN‐20 on day 2 post transfection. After 5 days, the culture supernatant was clarified harvested using centrifugation at 1500 *g*, 20 min at 4°C and cell debris was removed by filtration through a 0.45‐μm filter prior to Ni‐NTA chromatography. Samples buffered with PBS were loaded onto a PBS‐equilibrated HisTrap Excel column (Cytiva) equilibrated with PBS. After rinsing the column with PBS containing 20 mM imidazole, pH 7.5, the protein of interest was eluted using a gradient of 20–500 mM imidazole, pH 7.5, in five column volumes. Eluted fractions were analyzed using SDS‐PAGE on 4%–12% Bis‐Tris gradient gels (NuPAGE, Thermo Fisher Scientific), run in MES buffer at 200 V for 35 min, fixed and stained with Coomassie blue (Novex Blue Colloidal staining kit, Thermo Fisher Scientific). RBD monomers and dimers were separated by preative size exclusion chromatogparraphy using a HiLoad 16/600 Superdex 200 pg column (Cytiva) and eluted with PBS. Protein‐containing fractions were dialysed against at least a 100‐fold excess of PBS at 4°C overnight in Snakeskin dialysis tubing with MWCO of 10,000 Da (Thermo Fisher Scientific), quantified using A_280_ as determined by Nanodrop 2000c analysis (Thermo Fisher Scientific) and stored at –80°C until use.

### 2.3 Peptide and glycan analyses by mass spectrometry

Purified proteins were S‐alkylated with iodoacetamide after reduction with DTT and then digested with LysC (Roche)/GluC (Promega) or trypsin (Promega). For assessment of the reactive state of individual cysteine residues, proteins were reacted with N‐ethylmaleimide prior to treatment with DTT. Samples were analyzed using a maXis 4G ETD QTOF mass spectrometer (Bruker) as described^[^ ^10^ ^]^ (details in Methods, Supporting Information).

### 2.4 Biophysical analyses

#### 2.4.1 Size exclusion chromatography (SEC)

SEC‐HPLC was performed on a Shimadzu LC‐20A Prominence system equipped with a diode array detector with a Superdex 200 Increase 10/300 GL column (Cytiva), in PBS with 200 mM NaCl at a constant flow rate of 0.75 ml min^–1^. For analysis 20 μg protein at about 1 mg ml^–1^ were loaded. Column calibration was performed with a set of molecular mass standards ranging from 1.3 to 670 kDa (Bio‐RAD).

#### 2.4.2 Hydrophobic interaction chromatography (HIC)

Ten microgram protein were injected onto a Proteomix HIC Ethyl NP5 column (5 μm non‐porous, 4.6 × 100 mm) (Sepax Technologies Inc.) with a Proteomix HIC Ethyl NP5 guard column (5 μm, 4 mm × 10 mm), and the chromatographic run was performed at a flow rate of 1 ml min^–1^. First the column was equilibrated in 100% mobile phase A (25 mM Tris/HCl, pH 7.5, 1.5 M ammonium sulphate). After sample injection in 2.5 ml of 100% mobile phase A, the proteins were eluted with a linear gradient from 0% B to 100% B (25 mM Tris/HCl, pH 7.5, 20% v/v isopropanol) in 15 ml. After the application of 5 ml of 100% B, the column was re‐equilibrated with a gradient from 0% A to 100% A in 10 ml, and rinsed with 21.5 ml 100% A.

#### 2.4.3 Differential scanning calorimetry (DSC)

A MicroCal PEAQ‐DSC Automated system (Malvern Panalytical) was used to analyze 40 μM protein solutions in PBS at pH 7.5. The proteins were heated from 20°C to 100°C at a rate of 1°C/min, cooled in situ and re‐heated to obtain the baseline for subtraction from the first scan. All measurements were performed in duplicates with proteins from different expression batches. Fitting was done with Origin 7.0 for DSC software using the non‐2‐state transition model.

#### 2.4.4 Accelerated stability assays

SEC‐purified monomeric RBD preparations were diluted to 50 μg ml^–1^ in PBS and incubated for 7 days at 25°C. One hundred microliters of the samples were then analyzed with SEC‐HPLC as described above. Additionally, 20 μl of each sample was analyzed by SDS‐PAGE as described above.

### 2.5 Reactivity with convalescent sera

COVID‐19 sera utilized in the Luminex serotest included excess SARS‐CoV‐2‐convalescent and acute sera from non‐hospitalised blood donors (*n* = 124) collected before mid of June 2020. Ninety‐six of these sera were deidentified excess samples from routine SARS‐CoV‐2 serodiagnosis using a seven‐plex bead‐based Luminex‐FlexMap SARS‐CoV‐2 serotest. Control sera (*n* = 210) used in the Luminex assay were obtained from the Austrian Red Cross blood donor bank and were drawn in 2014. The use of excess sera without explicit consent and the study protocol were approved by the Ethics Committee of the city of Vienna (EK 20‐179‐0820).

Additional 28 residual sera (utilized in the Luminex assay as well as in the ELISA) were from unique patients or convalescent donors with (previous) SARS‐CoV‐2 infection collected from routine clinical examinations and were deposited at the MedUni Vienna Biobank. All individuals provided written informed consent for their samples to be added to a biobank and to be used for biomedical research/methods evaluation and the study protocol was approved by the ethics committee of the Medical University of Vienna (EK 1424/2020). Luminex‐ and ELISA‐based serological tests as well as statistical analysis of serological tests were performed as described previously^[^ ^10^ ^]^ (details in Methods, Supporting Information).

## 3 RESULTS

### 3.1 Design, production, and purification of RBD variants

**FIGURE 1:**
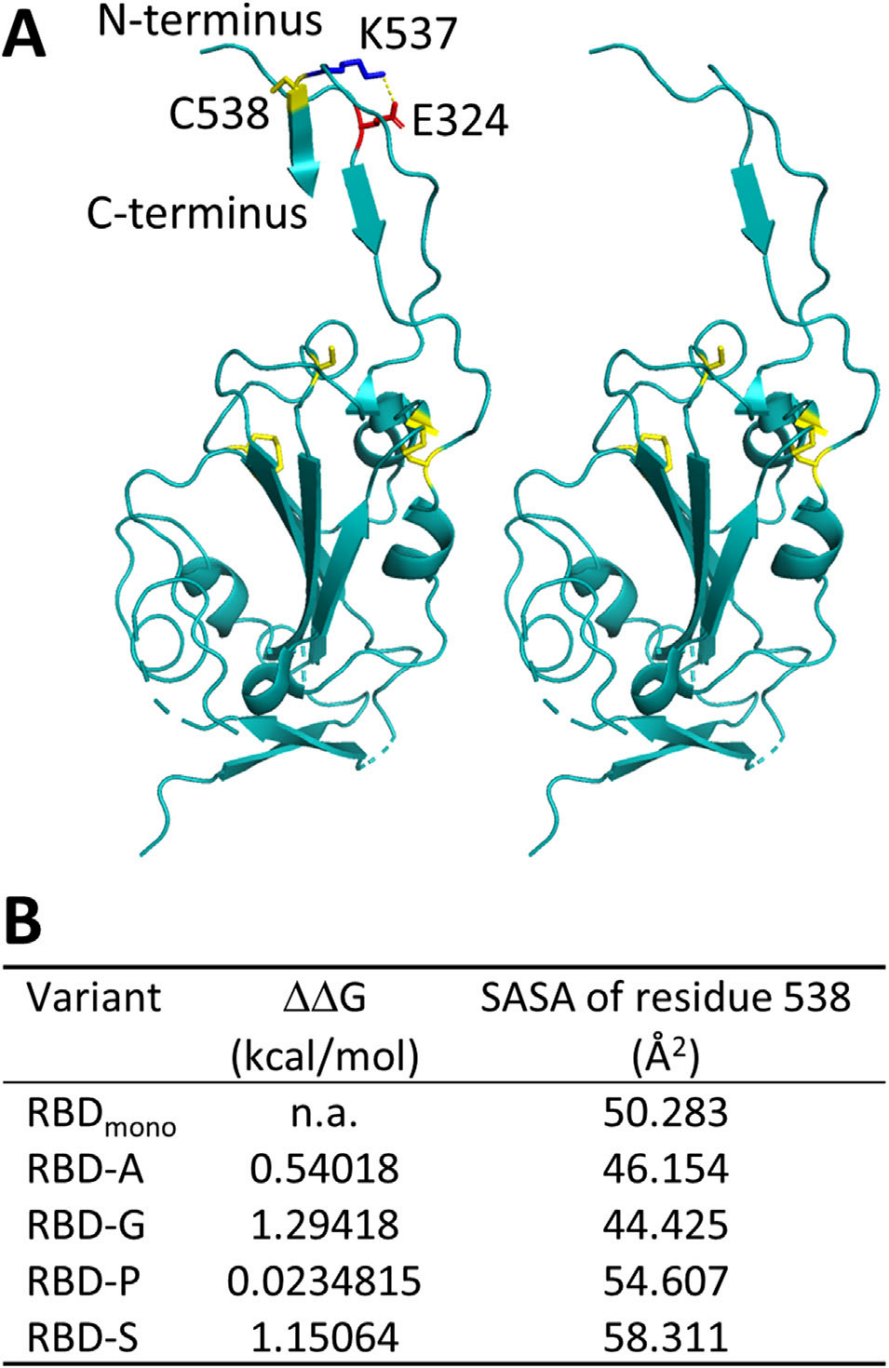
Molecular design of SARS‐CoV‐2 RBD variants: (A) structural models, left panel: wild‐type RBD (319‐541) with disulfide bridges and C538 in yellow and the salt bridge between E324 (red) and K537 (blue) highlighted; right panel: truncation mutant tRBD (319‐537). The figure was prepared with PyMOL (Schrödinger LLC) using RBD coordinates from PDB:6VXX, (B) stability changes of point mutations and the calculated SASA of residue 538 determined for the individual mutants. n.a., not applicable

**FIGURE 2:**
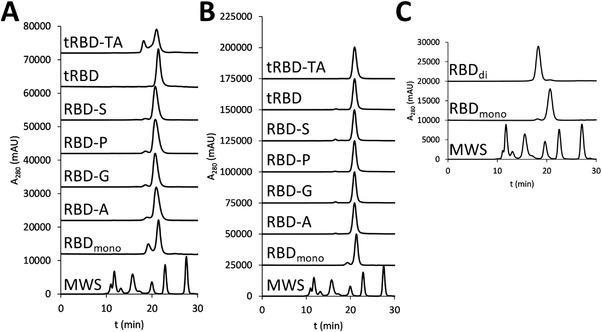
HPLC‐SEC profiles of RBD variants: (A) before SEC‐based purification, (B) after SEC purification, (C) comparison of wild‐type RBD (319‐541) monomer and dimer after SEC‐based purification. MWS: molecular weight standard (from left to right: 670, 158, 44, 17, and 1.3 kDa). tRBD: truncated RBD (319‐537); RBD_mono_: monomeric wild‐type RBD (319‐541); RBD_di_: dimeric wild‐type RBD (319‐541)

The RBD construct most extensively described in the literature – RBD (319‐541) – tends to form dimers when expressed in mammalian and insect cells as well as in plants.^[^ ^1^, ^10^ ^]^ It has been noted that this could be due to the presence of an unpaired cysteine residue (C538) close to the C‐terminus of the protein fragment.^[^ ^22^, ^23^ ^]^ We used computational modeling to design a truncated RBD variant and single amino acid substitution mutants (Figure 1A and Table S2, Supporting Information). The calculated thermodynamic stability of the resulting proteins and the SASA of the modified residues deviated only minimally from the wild‐type protein (Figure 1B). Furthermore, we mutated T323 to alanine to ablate the major O‐glycosylation site. All truncated and mutated constructs could be expressed in HEK293‐6E cells with yields exceeding 40 mg L^–1^, which compares with the amounts obtained with wild‐type RBD (319‐541) in transient expression systems^[^ ^1^, ^10^, ^24^ ^]^ and from stably transfected cell lines,^[^ ^22^ ^]^ although yields of up to 90 mg L^–1^ from transient expressions have been achieved after transfection optimization.^[^ ^9^ ^]^ While the native signal peptide of the SARS‐CoV‐2 was initially used for expression of RBD (319‐541), we later replaced it with the signal peptide of human heat stable alkaline phosphatase 1 and found that the expression level was not affected by the choice of the secretion signal. Hence, we used the signal peptide of alkaline phosphatase for all other constructs. After purification by metal‐chelate affinity chromatography, the proportion of dimer in the preparations of the mutants was consistently lower (3%–7%) than observed for the wild‐type 319–541 variant, where it amounted to 20%–25% (Figure 2A). Higher oligomers were observed in some preparations of RBD (319‐541) in very low amounts and were therefore not further characterized. The tRBD‐TA construct, in which the O‐glycosylation site at T323 was mutated, contained 25%–28% dimer (Figure 2A). Importantly, the dimeric forms could be effectively separated from the respective monomeric antigens by preparative SEC resulting in over 95% monodisperse species for all proteins tested (Figure 2B and C).

### 3.2 Glycan analysis

**FIGURE 3:**
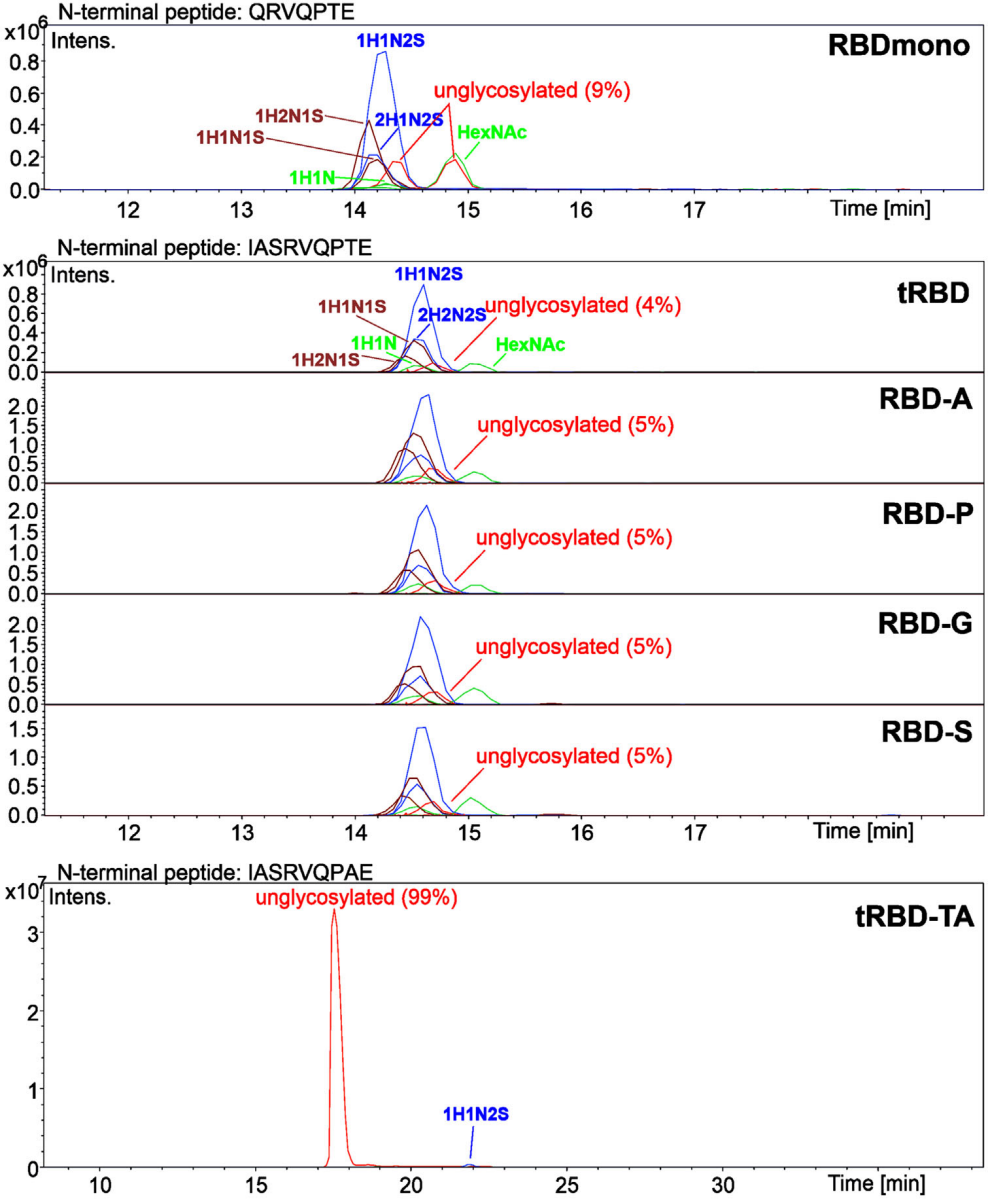
O‐glycan analysis of RBD variants by mass spectrometry. The N‐terminal peptide contains a mixture of different O‐glycans attached to T323, which consist of up to two hexose (H), N‐acetylhexosamine (HexNAc, N) and N‐acetylneuraminic acid (S) residues. Intens., ion intensity

Peptide mapping by mass spectrometry resulted in near complete coverage and confirmed the integrity of the N‐ and C‐termini of the proteins (Figure S1, Supporting Information). Furthermore, the N‐glycosylation patterns of all variants were comparable, with most detected compositions corresponding to complex‐type N‐glycans that are typically found on HEK293‐derived glycoproteins (Figures S2 and S3, Supporting Information). Wild‐type RBD (319‐541), tRBD, and the C538 mutants were found to be O‐glycosylated (> 90%) at T323, whereas O‐glycans were absent (< 1%) in the case of tRBD‐TA (Figure 3). Interestingly, the additional potential N‐glycosylation site N536 in the mutant C538S was only scarcely utilized as less than 3% of the molecules contained N‐glycans at this site (data not shown).

### 3.3 Biophysical characterization and ACE2‐binding

**FIGURE 4:**
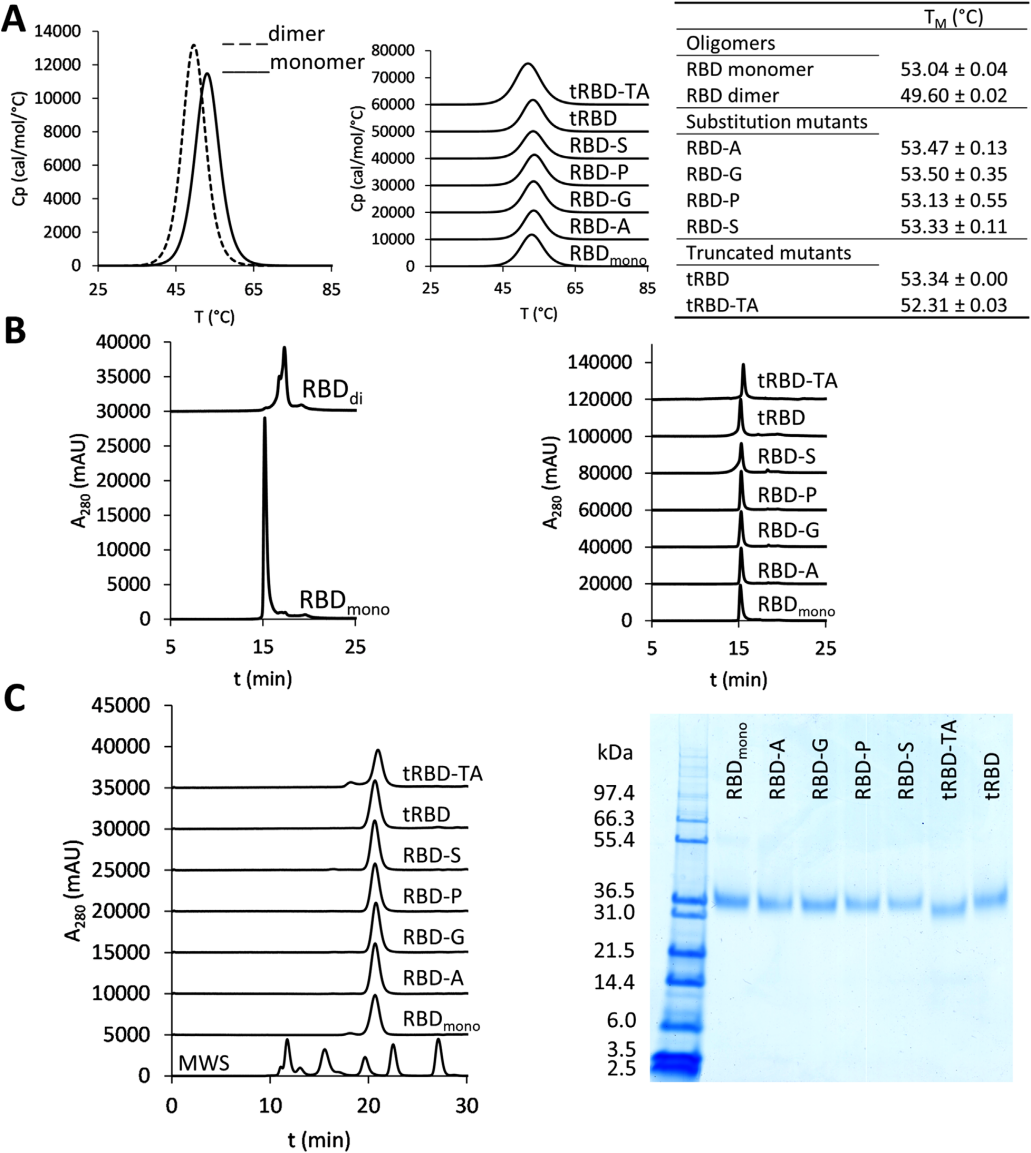
Biophysical properties of RBD variants: (A) Thermal unfolding of RBD variants measured with DSC: left panel: overlay of endotherms of monomeric and dimeric wild‐type RBD (319‐541), central panel: thermal unfolding of RBD mutants and monomeric wild‐type RBD (RBD_mono_) (Cp, heat capacity), right panel: transition midpoints (*T*_M_) of RBD variants (mean ± SEM of two independent experiments), (B) HIC profiles of RBD variants: left panel: monomeric and dimeric wild‐type RBD (319‐541), right panel: RBD substitution and truncation mutants and RBD_mono_, (C) Accelerated stability assays of RBD variants (samples were incubated for 7 days at 25°C prior to analysis): left panel: HPLC‐SEC profiles (MWS: molecular weight standard – from left to right: 670, 158, 44, 17, and 1.3 kDa), right panel: a non‐reducing SDS‐PAGE, with molecular mass standard in the first lane on the left

Testing of the thermal stability of the RBD mutants was performed with DSC. Interestingly, the midpoint transition temperature (*T*_M_) of the SEC‐purified dimeric wild‐type RBD (49.6°C) was considerably lower than that of monomeric RBD (53.0°C), indicating partial misfolding (Figure 4A) and was similar as determined for a CHO‐expressed cysteine‐linked dimer (48.4°C).^[^ ^25^ ^]^ For most SEC‐purified monomeric proteins, a single transition at about 53°C was observed (Figure 4A). Only the *T*_M_ of tRBD‐TA (52.3°C) was slightly below that of wild‐type RBD (319‐541) and the other mutant variants.

In HIC, dimeric RBD eluted in multiple peaks at later time points than monomeric RBD (Figure 4B). This indicates the presence of a mixture of conformational states with a variable level of exposed hydrophobic residues, which probably results from misfolding and aggregation during the expression process. All SEC‐purified monomeric RBD mutants eluted at the same time, indicating a similar degree of hydrophobicity. Consistent with the absence of the hydrophilic O‐glycan attached to T323, elution of tRBD‐TA was delayed by about 1 min (Figure 4B). Interestingly, the truncated variant tRBD displayed a broader elution peak. This could arise from microheterogeneity due to higher structural flexibility, which is possibly linked to inefficient formation of the salt bridge between the C‐terminal residue K537 and E324 in this construct. Peak broadening was even more pronounced in the case of the RBD variant C538S, which contains an infrequently used additional N‐glycosylation site at N536. It was encouraging to find that the stability of most SEC‐purified monomeric RBD variants was not compromised by an incubation for 7 days in PBS at 25°C as determined by SEC‐HPLC and SDS‐PAGE analysis (Figure 4C). This temperature was chosen as temperature‐induced unfolding already starts at 37°C (Figure 4A and B). The only exception was tRBD‐TA, where 10% of the protein dimerized during the treatment. The faster migration of tRBD‐TA on SDS‐PAGE as compared to the other RBD variants could be due to the absence of O‐glycosylation. Interestingly, the unpaired cysteine residue C538 of wild‐type RBD (319‐541) did not promote dimerization of the purified monomeric protein upon prolonged incubation. In both dimeric and monomeric wild‐type RBD (319‐541), C538 was < 10% reactive with thiol probes in its unreduced state (Figure S4, Supporting Information), which could result from oxidation or capping by endogenous compounds such as glutathione during protein synthesis and secretion.

Most SEC‐purified monomeric RBD variants bound to immobilized ACE2‐Fc, prepared as described recently,^[^ ^26^ ^]^ with an affinity of 20–30 nM (Figure S5, Supporting Information, experimental details in Supplementary Methods). Hence, none of the introduced truncations or cysteine mutations affected the proper folding of the RBM. However, SEC‐purified dimeric wild‐type RBD (319‐541) bound much stronger to ACE2‐Fc as documented by a fast association, a slow dissociation and an apparent K_D_ of 0.8 nM.

### 3.4 Seroreactivity of RBD variants

**FIGURE 5:**
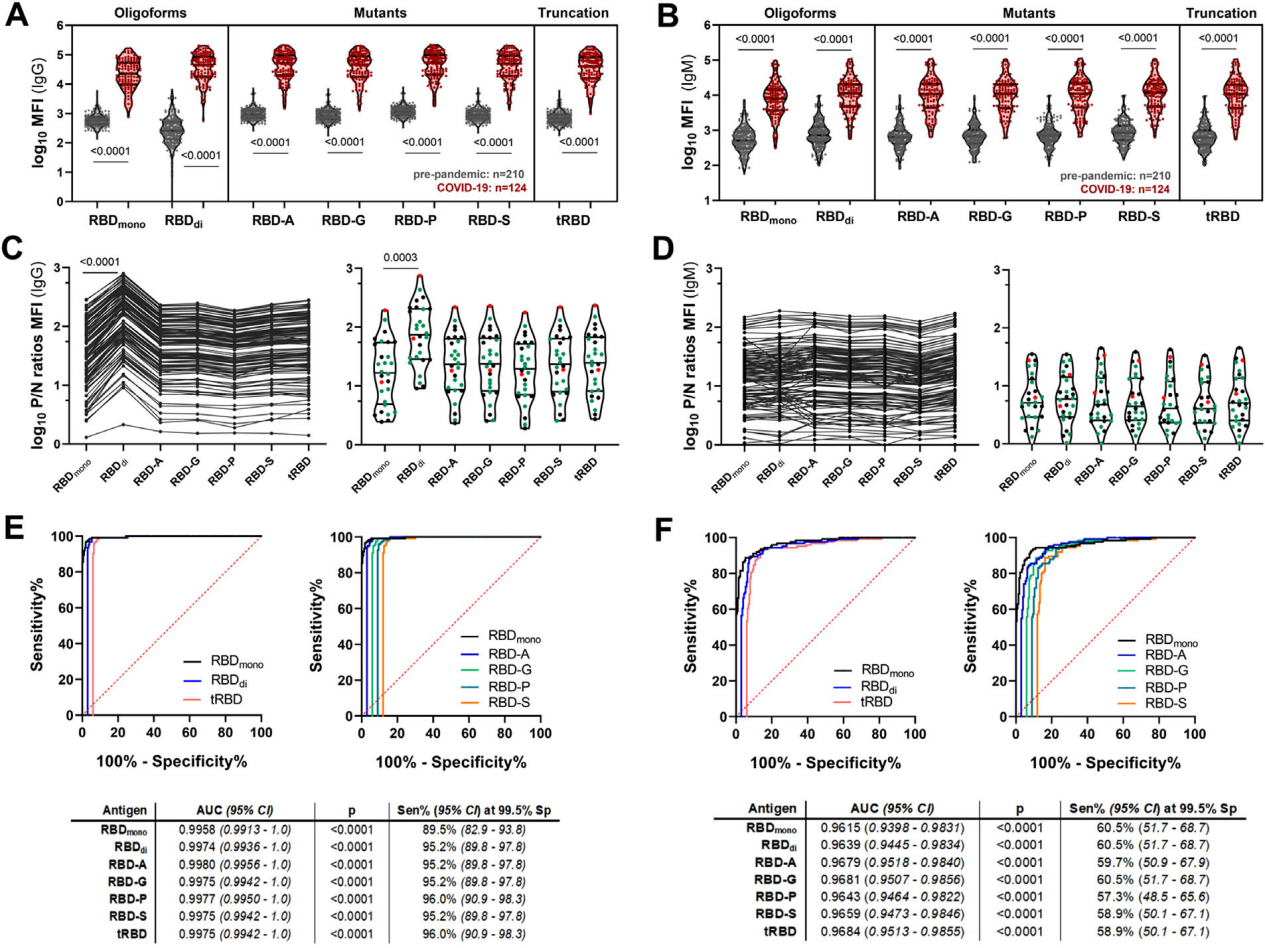
Reactivity of SARS‐CoV‐2 convalescent sera with RBD variants. (A and B) A bead‐based multiplex assay was performed with 124 sera from individuals with previous SARS‐CoV‐2 infection and 210 pre‐pandemic sera. Violin plots show the IgG (A) and IgM (B) immunoreactivity of individual sera as median fluorescence intensity (MFI). Lines indicate the median and quartiles. A nonparametric two‐tailed Mann‐Whitney U‐test was used to compare group medians of pre‐pandemic and convalescent sera. (C and D) The positive/negative (P/N) ratios give the IgG (C) or IgM (D) MFI readout of individual sera in comparison to the group median of the pre‐pandemic cohort. Individual sera are color‐coded as per the course of disease (green: asymptomatic and mild; black: moderate; red: severe), (E and F) Overlays of the areas under the receiver‐operating characteristic curve (ROC) for the IgG (E) and IgM (F) seroreactivity of all RBD variants. To ease visualization, ROC curves were horizontally nudged in respect to the ROC curve of RBD_mono_. Abbreviations: AUC, area under the curve; CI, confidence interval; Sen, sensitivity; Sp, specificity

Except tRBD‐TA (which was excluded from further studies due to its propensity to dimerize and the stability issues outlined above), all RBD variants were assayed for IgG immunoreactivity with a panel of convalescent COVID‐19 sera (*n* = 124) and pre‐pandemic human sera collected prior to 2018 (*n* = 210) using the Luminex platform (Figure 5). Despite obtaining very heterogeneous seroresponses within the cohorts, monomeric and dimeric RBD (319‐541), tRBD as well as all RBD mutants allowed for excellent discrimination between convalescent and control sera (*p* < 0.0001), with both IgG and IgM‐based assays (Figure 5A and B). Similar results were obtained when a subset of the COVID‐19 sera (*n *= 28) was analyzed by ELISA (Figure S6, Supporting Information). To analyze the seroprofiles between the antigens in more detail we calculated the ratios of the MFI readouts of individual COVID‐19 sera relative to the median MFI readout obtained with sera from the control cohort (positive/negative [P/N] ratios) and group medians were compared. Readouts with positive sera were 47‐ to 58‐fold higher than with control sera using monomeric RBD (319‐541), tRBD and RBD mutants as diagnostic antigens. The ratio was even higher when using dimeric RBD (178‐fold; *p* < 0.0001, Figure 5C). No significant differences between the groups were observed with the IgM‐based serotest (Figure 5D). For a small subset of the COVID‐19 sera (*n* = 28) we had data about the self‐assessed course of disease available, which allowed us to color‐code these data accordingly. For these sera, we observed that the magnitude of the IgG or IgM responses were very heterogeneous within the disease groups and did not reflect the severity of illness (Figure 5C and D, right panels). Despite the described higher median ratio in MFI readouts between positive and negative sera with dimeric RBD, the overall diagnostic performances were comparable, as demonstrated by the excellent area under the curve (AUC) values with all tested antigens (IgG: all > 0.99, IgM: all > 0.96, Figure 5E and F). It was astonishing to see that the marginal reduction in AUC values for the IgM‐based serotests resulted in a drastic decrease in sensitivities at the pre‐defined cutoff of 99.5% specificity (IgG: range 89.5%–96.0% vs. IgM: range 57.3%–60.5%).

## 4 DISCUSSION

The SARS‐CoV‐2 RBD is a key antigenic component of protein‐based vaccine candidates as well as diagnostic tests.[^1^,^10^,^11^] The tendency of RBD to form dimers has already been noticed at a very early stage of its structural and functional characterization.^[^ ^9^, ^27^, ^28^, ^29^ ^]^ Nonetheless, the molecular features driving RBD dimerization have remained unclear so far. We and others have observed that C‐terminally truncated RBD variants such as tRBD are less prone to dimerization and attributed this to the absence of the unpaired cysteine residue C538 in these constructs.^[^ ^10^, ^22^, ^23^, ^30^ ^]^ However, removal of C538 by truncation could concomitantly increase the flexibility of K537 and thus interfere with the formation of a stabilizing salt bridge between K537 and E324.^[^ ^12^ ^]^ We have therefore tested whether substitution of C538 with an uncharged amino acid of similar size (A, G, P, or S) would be a more suitable approach to reduce RBD dimerization while preserving its structural integrity. Our data indicate that tRBD and all four C538 mutants tested display comparable biophysical and seroreactive properties and do not show differences in ACE2‐binding. Likewise, dimer formation was reduced to a similar extent. However, deletion or substitution of C538 is not sufficient to completely abolish RBD dimerization: the residual propensity of RBD variants lacking C538 to form dimers is particularly pronounced after the introduction of stability‐enhancing mutations.^[^ ^27^ ^]^ It is of note that all these mutations (I358F, Y365W, V367F, F392W) increase the hydrophobicity of the protein. This could promote its tendency to aggregate via interactions between hydrophobic residues that are transiently exposed during protein folding and quality control in the endoplasmic reticulum,^[^ ^29^ ^]^ consistent with our observation that C538 is essentially unreactive once RBD has passed through the secretory pathway.

We have demonstrated that even small amounts of residual dimeric RBD (< 5%) can strongly affect kinetic studies of its interactions with ACE2 or other ligands. Interestingly, designed tandem repeat dimeric RBD resembles the monomer in ACE2 binding.^[^ ^31^ ^]^ The stronger interaction of naturally formed dimer determined here can reflect differences in the spacing between the RBMs, or be a consequence of the bivalent nature of the ACE2‐Fc ligand used in our study. Naturally formed dimeric RBD appears structurally impaired, judging from its lower thermostability as well as the considerable heterogeneity of the SEC‐purified dimer preparations in HIC analysis. This may contribute to differences in the seroreactivity of monomeric and dimeric RBD forms. Indeed, it has been already demonstrated in pre‐clinical mouse studies that RBD dimer preparations can be more immunogenic than the corresponding monomer.^[^ ^31^ ^]^ Variable dimer content could modify the immune response to RBD‐based vaccine formulations and it is hence crucial to closely monitor the oligomeric state of RBD‐based vaccines, particularly if the protein contains an unpaired cysteine resulting from the design of an isolated domain as in the case of RBD (319‐541).

Under our culture conditions, up to 25% RBD in HEK293 supernatants was dimeric. Even higher dimer contents were observed after up‐scaled production in HEK293‐derived cell lines using stirred bioreactors and wave bags.^[^ ^9^ ^]^ Dimer removal for the generation of homogeneous monomeric RBD preparations not only necessitates an additional purification step during vaccine manufacturing, up to 50% of the recombinant protein yield may need to be discarded. Therefore, RBD constructs that are less prone to dimerization are a preferred alternative for achieving high product yields. Recent studies reveal that also the choice of HEK Expi293F cells as expression host^[^ ^9^ ^]^ as well as virus‐mediated DNA delivery instead of plasmid transfection results in less dimer.^[^ ^32^ ^]^ Separation of monomeric and dimeric RBD is usually accomplished by preparative SEC, which poses a challenge for upscaling of the purification procedure.^[^ ^11^, ^28^ ^]^ We have found that monomeric and dimeric RBD can be also effectively separated by HIC, which is superior to SEC with respect to its scalability and thus easier to implement in industrial production, and the feasibility of HIC‐based RBD‐purification steps in its large‐scale downstream processing has been already demonstrated.^[^ ^33^ ^]^

Elimination of C538 by C‐terminal truncation or a point mutation did not result in major changes in the immunoreactivity of RBD with sera from SARS‐CoV‐2‐infected individuals when utilized as diagnostic antigen in Luminex bead‐based tests as well as ELISA assays. We used COVID‐19 serum samples from non‐hospitalized individuals with disease symptoms ranging from asymptomatic to severe, which is reflected by the heterogeneity in the observed seroreactivities. Sera was drawn maximum 4 months after acute SARS‐CoV‐2 infection. Within such a short time frame, an earlier onset of antibody waning makes IgM responses usually much more dynamic than IgG responses.^[^ ^34^ ^]^ Interestingly, control sera displayed a unique seroreactivity pattern with dimeric RBD, suggesting an antigen‐specific effect. Our HIC data suggests that the RBD dimer is a conformationally heterogeneous species with a certain degree of misfolding. This may have caused higher unspecific binding with control sera and the detection antibody, which after blank correction ultimately yielded a broader spectrum of fluorescence readouts than with other antigens. While our data suggest that the usually unspecified dimer content of commercial RBD preparations will not affect the overall performance of seroassays, dimer presence could affect the long‐term stability of RBD and thus diagnostic performance after extended storage.

It is well established that proper N‐glycosylation of N331 and N343 is important for correct folding of the RBD.^[^ ^30^, ^31^, ^35^ ^]^ Although it has been reported before that RBD also contains O‐glycans,^[^ ^18^, ^36^, ^37^ ^]^ the functional relevance of RBD O‐glycosylation has been hitherto not addressed In our RBD preparations derived from HEK293‐6E cells, T323 is consistently O‐glycosylated in an almost quantitative manner (> 90%). Other reports have also noted substantial O‐glycosylation of T323 in other monomeric S‐protein variants.^[^ ^36^, ^37^ ^]^ Interestingly, O‐glycosylation of T323 is far less pronounced in the context of full‐length S‐protein trimers,^[^ ^37^, ^38^, ^39^, ^40^ ^]^ indicating that this site is shielded upon trimer formation and thus not accessible to glycosylation enzymes once oligomerization has taken place. In RBD, T323 O‐glycosylation, characterized as disialyl Core 1 and Core 2 structures^[^ ^39^ ^]^ could be required to prevent oligomerization of RBD monomers by masking an otherwise aggregation‐prone hydrophobic peptide stretch or by balancing the overall charge of the protein. The mutation of T323 into alanine essentially eliminated O‐glycosylation of the N‐terminal RBD peptide and concomitantly increased the propensity of the protein to form dimers as well as lowered its thermal stability. Other studies of RBD mutants with N‐terminal truncations that removed T323 also report monomer loss upon incubation at room temperature.^[^ ^24^ ^]^ However, the capacity of RBD to interact with ACE2 was thereby not compromised, which is consistent with structural data demonstrating that T323 is not located in the proximity of the ACE2‐binding site.^[^ ^12^ ^]^

In conclusion, our study has revealed that the dimerization propensity of the most widely used SARS‐CoV‐2 S‐protein RBD variant encompassing amino acids 319–541 can be reduced by truncation or substitution of the unpaired cysteine residue C538 without compromising the biophysical properties and seroreactivity of the protein. Furthermore, we have shown that the O‐glycans attached to T323 also counteract RBD dimerization. These findings will support ongoing efforts to design improved RBD‐based vaccines and diagnostic reagents.

## CONFLICT OF INTEREST

The authors declare that the research was conducted in the absence of any commercial or financial relationships that could be construed as a potential conflict of interest.

## Supporting information



Supporting Information

## Data Availability

The data that support the findings of this study are available within this article and supporting information, and on request from the corresponding author.
