# Supplementary material for: Designed SARS‐CoV‐2 receptor binding domain variants form stable monomers
Source: Biotechnol J. 2022 Feb 3;17(5):2100422. doi: 10.1002/biot.202100422 (PMC9011732; doi:10.1002/biot.202100422)
Supplement: Supplementary file 1 — Supporting Information [file BIOT-17-0-s001.pdf]

Supporting Information

Designed SARS-CoV-2 receptor binding domain variants form stable monomers

Miriam Klausberger[1]*, Nikolaus F. Kienzl[2]*, Gerhard Stadlmayr[1,3]*, Clemens Grünwald-Gruber[4], Elisabeth Laurent[1,5], Katharina Stadlbauer[1,3], Florian Stracke[1,3], Klemens Vierlinger[6], Manuela Hofner[6], Gabriele Manhart[7], Wilhelm Gerner[8], Florian Grebien[7], Andreas Weinhäusel[6], Lukas Mach[2] and Gordana Wozniak-Knopp[1,3]

[1]Institute of Molecular Biotechnology, Department of Biotechnology, University of Natural Resources and Life Sciences (BOKU), Vienna, Muthgasse 18, 1190 Vienna, Austria

[2]Institute of Plant Biotechnology and Cell Biology, Department of Applied Genetics and Cell Biology, University of Natural Resources and Life Sciences (BOKU), Vienna, Muthgasse 18, 1190 Vienna, Austria

[3]Christian Doppler Laboratory for Innovative Immunotherapeutics, University of Natural Resources and Life Sciences (BOKU), Vienna, Muthgasse 18, 1190 Vienna, Austria

[4]Institute of Biochemistry, Department of Chemistry and BOKU Core Facility Mass Spectrometry, University of Natural Resources and Life Sciences (BOKU), Vienna, Muthgasse 18, 1190 Vienna, Austria

[5]BOKU Core Facility Biomolecular & Cellular Analysis, University of Natural Resources and Life Sciences (BOKU), Vienna, Muthgasse 18, 1190 Vienna, Austria

[6]Competence Unit Molecular Diagnostics, Center for Health and Bioresources, Austrian Institute of Technology, Giefinggasse 4, 1210 Vienna, Austria

[7]Institute of Medical Biochemistry, University of Veterinary Medicine, Veterinärplatz 1, 1210 Vienna, Austria

[8]Institute of Immunology, University of Veterinary Medicine, Veterinärplatz 1, 1210 Vienna, Austria

*equal contributions

Correspondence:

Gordana Wozniak-Knopp (protein engineering): Institute of Molecular Biotechnology, Department of Biotechnology, University of Natural Resources and Life Sciences, Muthgasse 18, A-1190 Vienna, Austria; e-mail: gordana.wozniak@boku.ac.at

Miriam Klausberger (immunoassays): Institute of Molecular Biotechnology, Department of Biotechnology, University of Natural Resources and Life Sciences, Muthgasse 18, A-1190 Vienna, Austria; e-mail: miriam.klausberger@boku.ac.at

Lukas Mach (glycosylation): Institute of Plant Biotechnology and Cell Biology, Department of Applied Genetics and Cell Biology, University of Natural Resources and Life Sciences, Muthgasse 18, A-1190 Vienna, Austria; e-mail: lukas.mach@boku.ac.at

Supplementary Methods.

Peptide and glycan analyses by mass spectrometry. Digested samples were loaded on a Thermo Acclaim PepMap300 RSLC C18 separation column (2 µm particle size, 150 x 0.075 mm) with a Thermo Acclaim PepMap µ-precolumn using 0.1% formic acid as the aqueous solvent. A gradient from 6% B (B: 80% acetonitrile) to 40% B in 30 min was applied, followed by a 10 min gradient from 40% B to 90% B that facilitates elution of large peptides, at a flow rate of 0.3 µL/min. Detection was performed with a Bruker maXis 4G ETD QTOF MS equipped with a captive spray source in positive ion, DDA mode (= switching to MS/MS mode for eluting peaks). MS-scans were recorded (range: 150-2200 Da) and the 6 highest peaks were selected for fragmentation. Instrument calibration was performed using an ESI calibration mixture (Agilent Technologies). Using Data Analysis (Bruker), raw data were converted into mgf files, which are suitable for performing a MS/MS ion search with MASCOT. The files were then searched against a database containing the target sequences.

Glycopeptides were identified as sets of peaks consisting of the peptide moiety and the attached N- or O-glycan varying in the number of N-acetylhexosamine, hexose, fucose and N-acetylneuraminic acid residues. The theoretical masses of these glycopeptides were determined using the monoisotopic masses of the respective amino acids and monosaccharides.

Biolayer interferometry (BLI) assays. ACE2-Fc was labelled with Sulfo-NHS-LC-biotin (Thermo Fisher Scientific) at a molar ratio of 1:5 for 30 min at 25 °C. Excess reagent was quenched by addition of Tris-HCl buffer (pH 7.4) to a final concentration of 3 mM. Biotinylated ACE2-Fc was purified using a PD-10 desalting column (Cytiva) according to the manufacturer's protocol. An Octet 96REDe (ForteBio)-based assay was used for determination of binding kinetics, using PBS supplemented with 0.05% (v/v) Tween-20 and 0.1% bovine serum albumin (BSA) as the assay buffer at 25 °C with the plate shaking at 1000 rpm. Biotinylated ACE2-Fc was immobilized on high-precision streptavidin tips (ForteBio) equilibrated in assay buffer at 6.5 µg/mL until a signal of 0.9 nm was achieved. RBD preparations in two-fold dilution series starting with 200 nM were allowed to bind to the immobilized receptor for 600 s at 25 °C. Dissociation was monitored for 300 s after transfer of the tips into assay buffer. Assay background was determined with an ACE2-Fc coated tip immersed in

assay buffer only. Each experiment was performed in triplicate. After background measurement subtraction, data were fitted with the 1:1 binding model, taking only the first 100 s of the dissociation phase into account. To obtain an apparent K_D for binding of RBD_{di} to ACE2-Fc only 100 s of the association phase could be taken into account.

Statistical analysis of serological tests. Raw data were corrected for blanks and assessed for normality of distribution and homogeneity of variances using the D'Agostino–Pearson omnibus test before statistical procedures. Differences in MFI (Luminex) or absorbance (ELISA) between convalescent and pre-COVID control sera were compared using unpaired, two-tailed Mann-Whitney U tests. Positive/Negative (P/N) ratios were calculated for positive sera in relation to the median of the pre-pandemic control cohort. P/N ratios were assessed for normality and were compared by a non-parametric Kruskal-Wallis test followed by a Dunn's post hoc test, with the monomeric RBD (319-541) group (RBD_{mono}) as reference. Diagnostic performance was analysed by area under the receiver operating characteristic (AUROC) curve analysis. Data on the diagnostic performance of the antigens and their cross-reactivity were analysed using GraphPad Prism Version 8.1.0 (GraphPad Software, San Diego, CA, USA).

Supplementary Table 1. Oligonucleotide primers used in this study.

Mutant proteins	Oligonucleotide
Truncated mutants	
tRBD_nhe1	acgtgctagc cgggtgcagc ccaccgaatc
tRBD_bste2	acgtggtgac cggtgctgga acctttgttc ttcacgagat tg
T323A mutation	
tRBD-TA	tagccgggtg cagcctgctg aatccatcgt gcgg
tRBD-TAa	ccgcacgatg gattcagcag gctgcacccg gcta
C538 substitutions	
RBD_C538A	ctcgtgaaga acaaagcagt gaacttcggt tcc
RBD_C538Aa	ggaaccgaag ttcactgctt tgttcttcac gag
RBD_C538G	ctcgtgaaga acaaaggcgt gaacttcggt tcc
RBD_C538Ga	ggaaccgaag ttcacgcctt tgttcttcac gag
RBD_C538P	ctcgtgaaga acaaacctgt gaacttcggt tcc
RBD_C538Pa	ggaaccgaag ttcacaggtt tgttcttcac gag
RBD_C538S	ctcgtgaaga acaaaagcgt gaacttcggt tcc
RBD_C538Sa	ggaaccgaag ttcacgcttt tgttcttcac gag

Supplementary Table 2. Amino acid sequences of RBD variants used in this study.

RBD (319-541):
QRVQPTESIVRFPNITNLCPFGEVFNATRFASVYAWNRKRISNCVADYSVLYNSASFSTFKCYGVSPTKLNDLCFTNV
YADSFVIRGDEVRQIAPGQTGKIADYNYKLPDDFTGCVIAWNSNNLDSKVGGNYNYLYRLFRKSNLKPFERDISTEIY
QAGSTPCNGVEGFNCYFPLQSYGFQPTNGVGYQPYRVVVLSFELLHAPATVCGPKKSTNLVKNKCVNFHHHHHH

RBD-A (Cys538->Ala):
IASRVQPTESIVRFPNITNLCPFGEVFNATRFASVYAWNRKRISNCVADYSVLYNSASFSTFKCYGVSPTKLNDLCFTN
VYADSFVIRGDEVRQIAPGQTGKIADYNYKLPDDFTGCVIAWNSNNLDSKVGGNYNYLYRLFRKSNLKPFERDISTEI
YQAGSTPCNGVEGFNCYFPLQSYGFQPTNGVGYQPYRVVVLSFELLHAPATVCGPKKSTNLVKNK**A**VNFGSSTGH
HHHHHHHG

RBD-G (Cys538->Gly):
IASRVQPTESIVRFPNITNLCPFGEVFNATRFASVYAWNRKRISNCVADYSVLYNSASFSTFKCYGVSPTKLNDLCFTN
VYADSFVIRGDEVRQIAPGQTGKIADYNYKLPDDFTGCVIAWNSNNLDSKVGGNYNYLYRLFRKSNLKPFERDISTEI
YQAGSTPCNGVEGFNCYFPLQSYGFQPTNGVGYQPYRVVVLSFELLHAPATVCGPKKSTNLVKNK**G**VNFGSSTGH
HHHHHHHG

RBD-P (Cys538->Pro):
IASRVQPTESIVRFPNITNLCPFGEVFNATRFASVYAWNRKRISNCVADYSVLYNSASFSTFKCYGVSPTKLNDLCFTN
VYADSFVIRGDEVRQIAPGQTGKIADYNYKLPDDFTGCVIAWNSNNLDSKVGGNYNYLYRLFRKSNLKPFERDISTEI
YQAGSTPCNGVEGFNCYFPLQSYGFQPTNGVGYQPYRVVVLSFELLHAPATVCGPKKSTNLVKNK**P**VNFGSSTGH
HHHHHHHG

RBD-S (Cys538->Ser):
IASRVQPTESIVRFPNITNLCPFGEVFNATRFASVYAWNRKRISNCVADYSVLYNSASFSTFKCYGVSPTKLNDLCFTN
VYADSFVIRGDEVRQIAPGQTGKIADYNYKLPDDFTGCVIAWNSNNLDSKVGGNYNYLYRLFRKSNLKPFERDISTEI
YQAGSTPCNGVEGFNCYFPLQSYGFQPTNGVGYQPYRVVVLSFELLHAPATVCGPKKSTNLVKNK**S**VNFGSSTGHH
HHHHHHG

tRBD:
IASRVQPTESIVRFPNITNLCPFGEVFNATRFASVYAWNRKRISNCVADYSVLYNSASFSTFKCYGVSPTKLNDLCFTN
VYADSFVIRGDEVRQIAPGQTGKIADYNYKLPDDFTGCVIAWNSNNLDSKVGGNYNYLYRLFRKSNLKPFERDISTEI
YQAGSTPCNGVEGFNCYFPLQSYGFQPTNGVGYQPYRVVVLSFELLHAPATVCGPKKSTNLVKNKGSSTGHHHHH
HHHG

tRBD-TA (Thr323 -> Ala):
IASRVQP**A**ESIVRFPNITNLCPFGEVFNATRFASVYAWNRKRISNCVADYSVLYNSASFSTFKCYGVSPTKLNDLCFTN
VYADSFVIRGDEVRQIAPGQTGKIADYNYKLPDDFTGCVIAWNSNNLDSKVGGNYNYLYRLFRKSNLKPFERDISTEI
YQAGSTPCNGVEGFNCYFPLQSYGFQPTNGVGYQPYRVVVLSFELLHAPATVCGPKKSTNLVKNKGSSTGHHHHH
HHHG

Supplementary Figure 1. Peptide mapping of the RBD variants used in this study. Potential N-glycosylation sites are highlighted in yellow.

RBD (319-541):

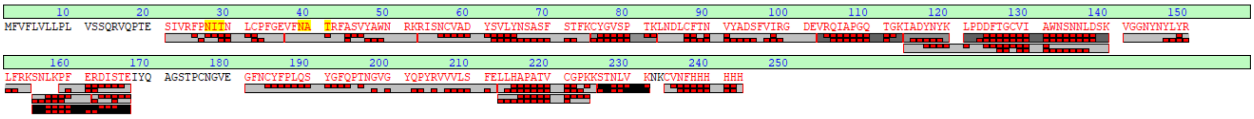

RBD-A (C538->A):

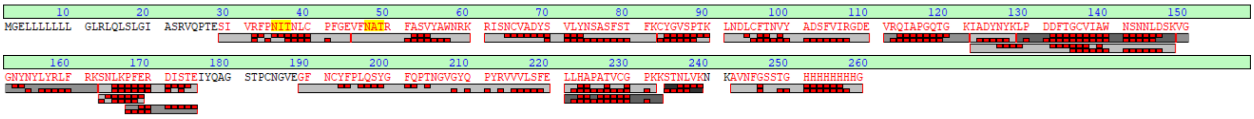

RBD-S (C538->S):

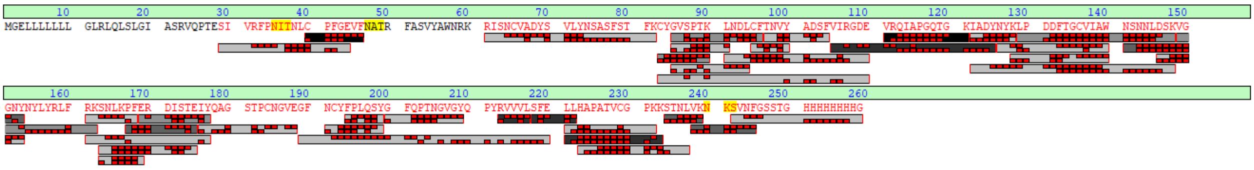

RBD-G (C538->G):

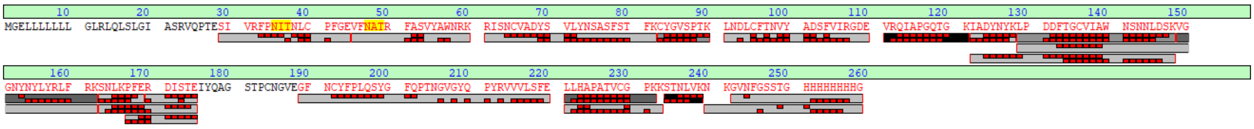

RBD-P (C538->P):

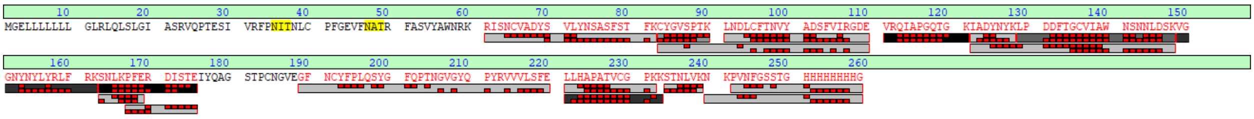

tRBD:

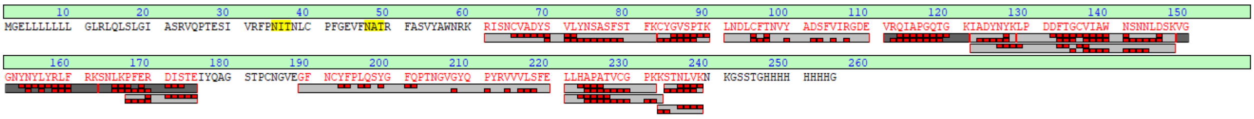

tRBD-TA (T323->A):

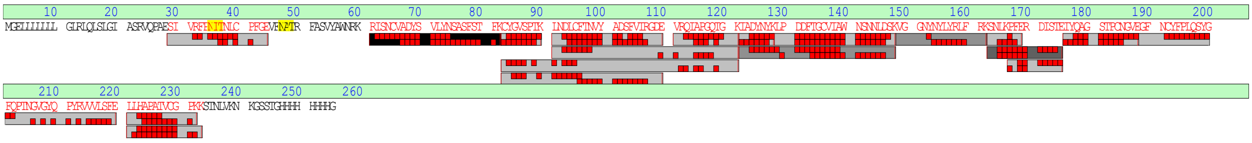

Supplementary Figure 2. Compositions of N-glycans attached to N-glycosylation site 1 (N331). F, fucose; H, hexose; N, N-acetylhexosamine; S, N-acetylneuraminic acid (S). Intens., ion intensity.

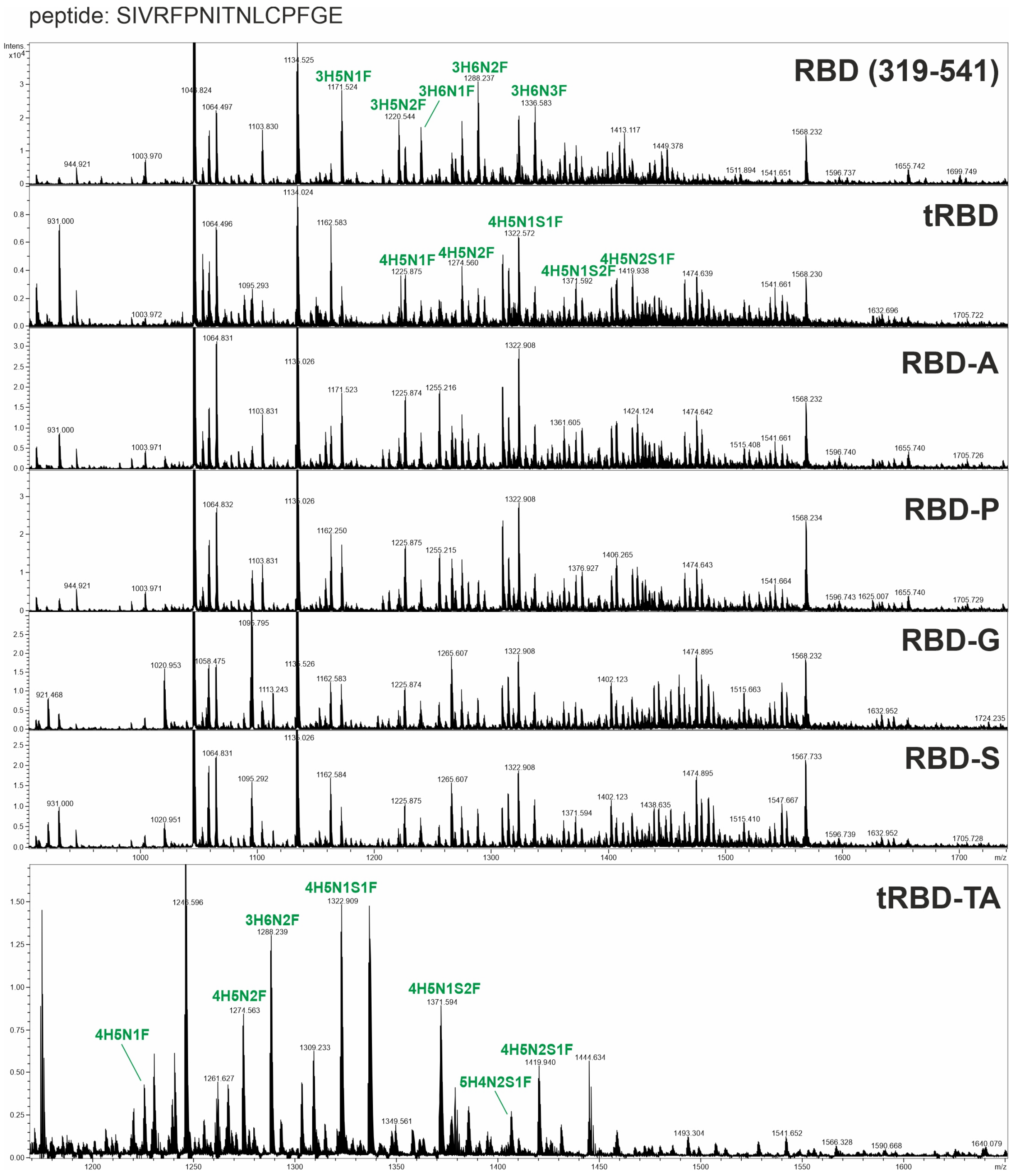

Supplementary Figure 3. Compositions of N-glycans attached to N-glycosylation site 2 (N343). F, fucose; H, hexose; N, N-acetylhexosamine; S, N-acetylneuraminic acid (S). Intens., ion intensity.

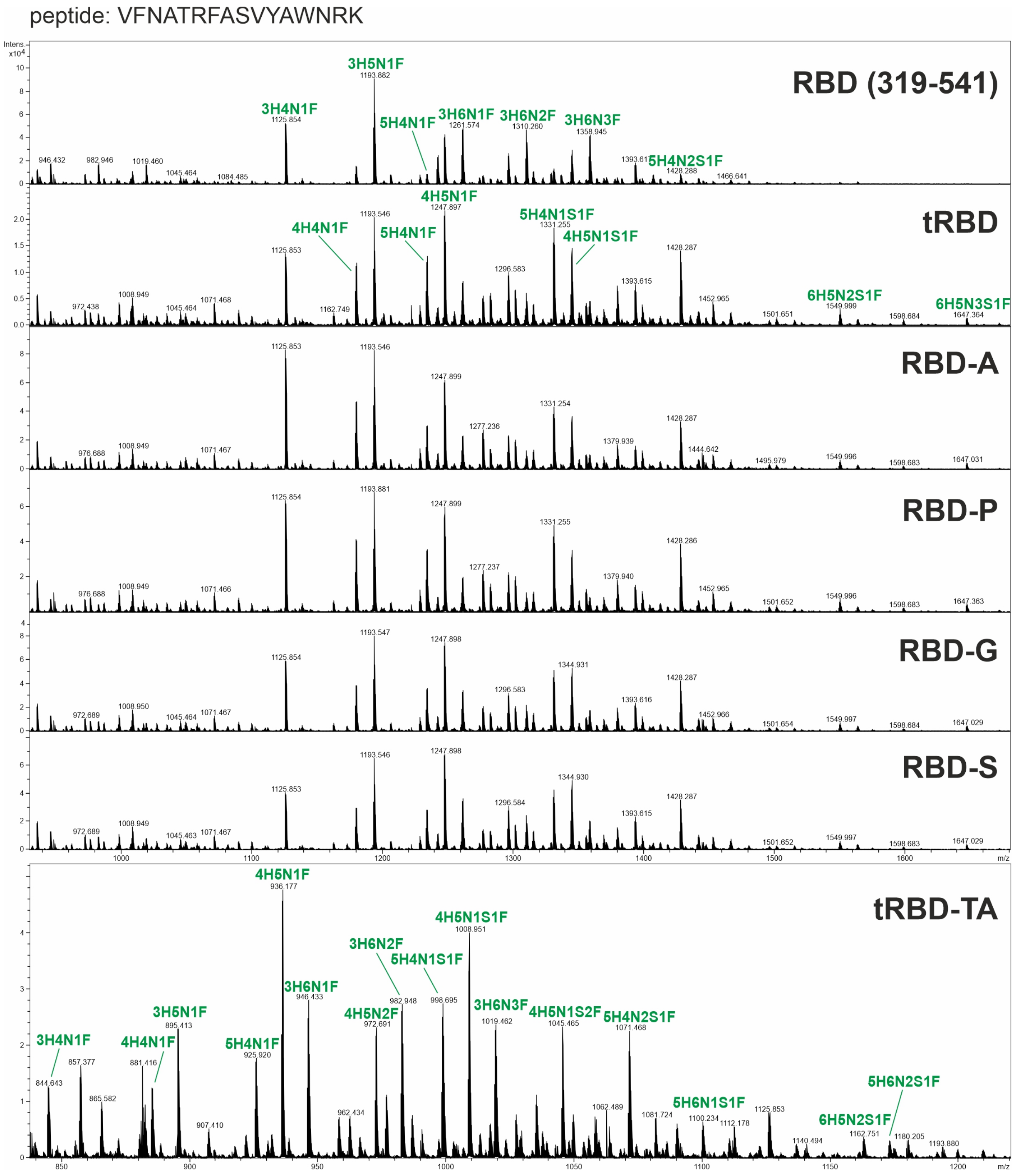

Supplementary Figure 4. Thiol reactivity of RBD residue C538. Purified monomeric and dimeric wild-type RBD (319-541) forms were first reacted with N-ethylmaleimide (NEM), then reduced and alkylated with iodoacetamide (IAM) prior to digestion with trypsin and analysis by mass spectrometry. Red traces, NEM-modified C-terminal tryptic peptide C^{538}VNFHHHHHH; black traces, IAM-modified C^{538}VNFHHHHHH. Intens., ion intensity.

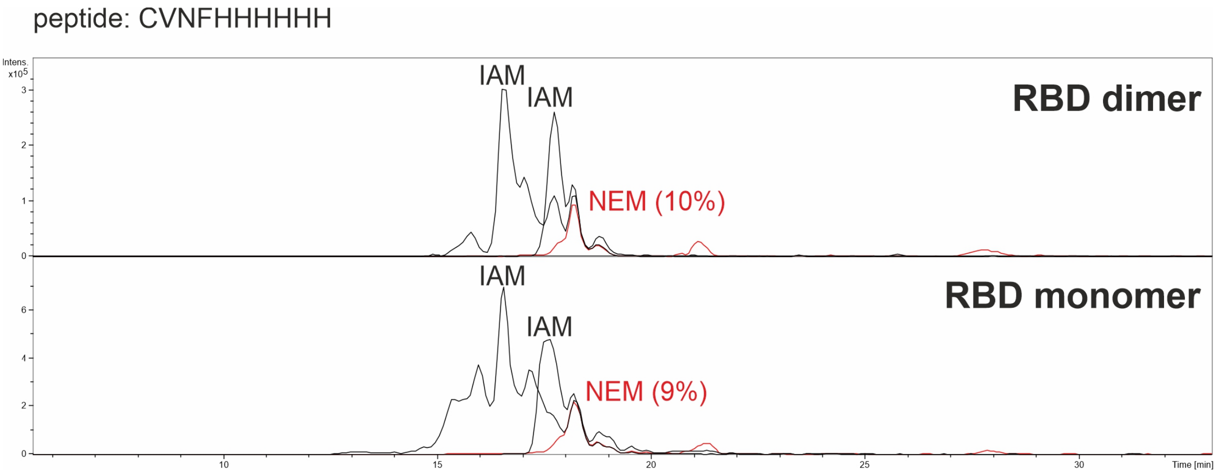

Supplementary Figure 5. Binding of SEC-purified RBD variants to immobilized ACE2-Fc as determined with biolayer interferometry. The RBD concentration range tested was in each case 6.25 – 200 nM. Affinity constants of RBD variants for binding to immobilized ACE2-Fc were determined by biolayer interferometry. Data represent the mean ± SEM of three independent experiments. Note the much slower dissociation kinetics of dimeric wild-type RBD (319-541) as compared to all monomeric RBD forms.

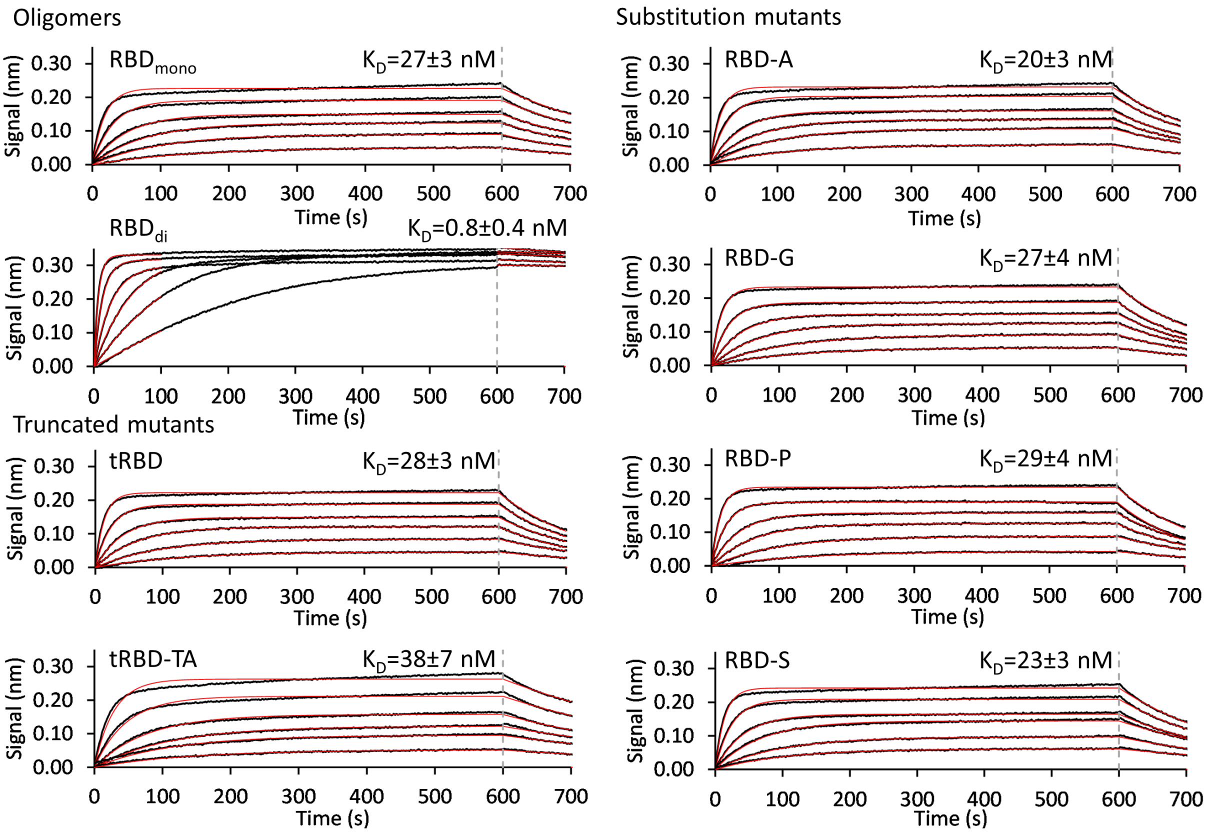

Supplementary Figure 6. Reactivity of convalescent COVID-19 sera (n = 31) and pre-COVID controls (n = 12-14) with different HEK293-produced RBD variants as determined by ELISA. (A) Violin plots representing the IgG immunoreactivity of individual sera as optical density (OD) at 450 nm. Individual data points correspond to the mean of three blank-corrected replicates. Lines indicate the median and quartiles. A nonparametric two-tailed Mann-Whitney U-test was used to compare group medians of pre-pandemic and convalescent sera, (B) Overlays of the areas under the respective receiver-operating characteristic curve (ROC) for the IgG seroreactivity of all RBD variants. To ease visualization, ROC curves were horizontally nudged in respect to the ROC curve of RBD$_{mono}$. AUC, area under the curve; CI, confidence interval.

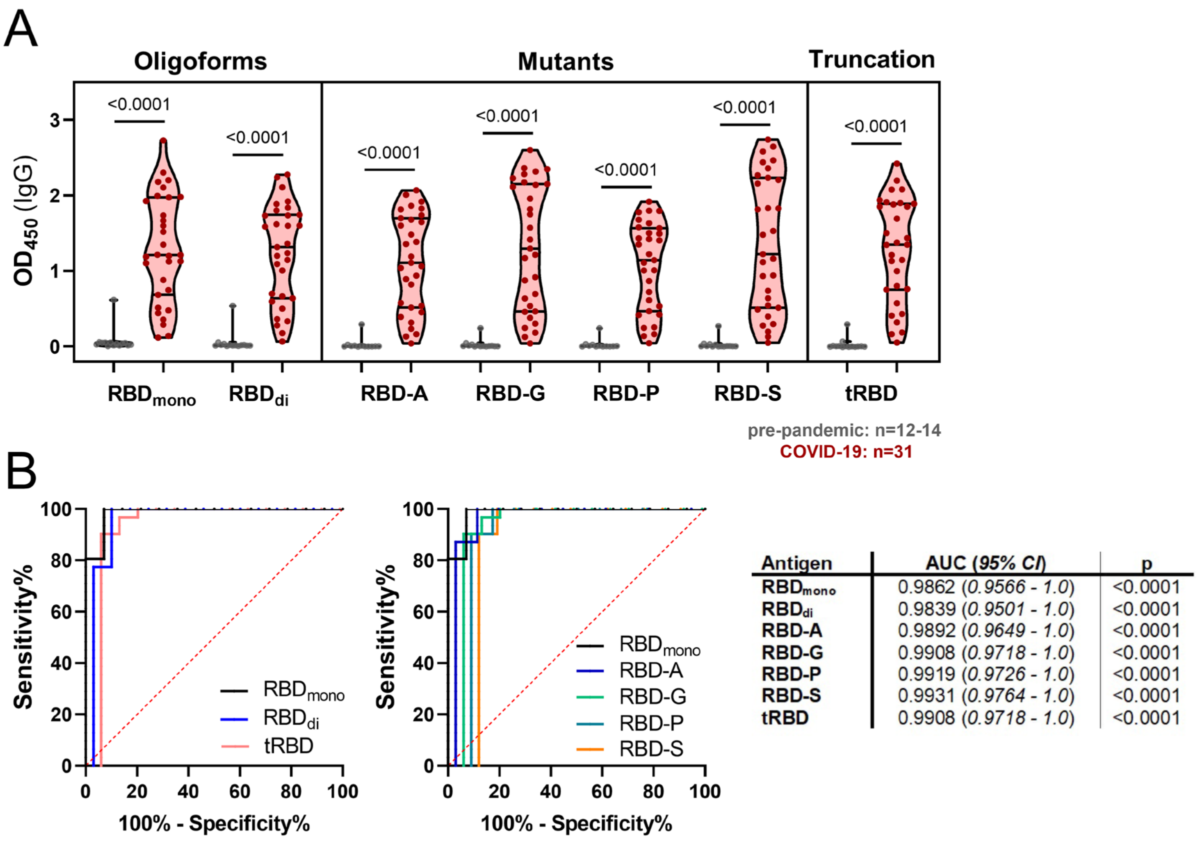

Antigen	AUC (*95% CI*)	p
**RBD$_{mono}$**	0.9862 (*0.9566 - 1.0*)	<0.0001
**RBD$_{di}$**	0.9839 (*0.9501 - 1.0*)	<0.0001
**RBD-A**	0.9892 (*0.9649 - 1.0*)	<0.0001
**RBD-G**	0.9908 (*0.9718 - 1.0*)	<0.0001
**RBD-P**	0.9919 (*0.9726 - 1.0*)	<0.0001
**RBD-S**	0.9931 (*0.9764 - 1.0*)	<0.0001
**tRBD**	0.9908 (*0.9718 - 1.0*)	<0.0001
